# Author response to: Comment on: Reducing the environmental impact of surgery on a global scale: systematic review and co-prioritization with healthcare workers in 132 countries

**DOI:** 10.1093/bjs/znad183

**Published:** 2023-06-19

**Authors:** Virginia Ledda, Aneel Bhangu, Dmitri Nepogodiev

**Affiliations:** NIHR Global Health Research Unit on Global Surgery, Institute of Applied Health Research, University of Birmingham, Birmingham, UK; NIHR Global Health Research Unit on Global Surgery, Institute of Applied Health Research, University of Birmingham, Birmingham, UK; NIHR Global Health Research Unit on Global Surgery, Institute of Applied Health Research, University of Birmingham, Birmingham, UK

*Dear Editor*

Thanks to Davies and McGain^1^ for their insightful comments. The Original Article represented an attempt to engage a global community of healthcare professionals to identify feasible, safe, and immediately actionable interventions to decarbonize operating theatres^2^. There was a focus on operating theatres because they are controlled environments involving the treatment of one patient at a time. This means that interventions can be carefully introduced and their effects can be closely monitored. Interventions proven to reduce carbon emissions whilst maintaining acceptable cost and safety profiles can then be scaled hospital-wide.

It is agreed that preventative healthcare and reduction of low-value surgery could significantly reduce the environmental impact of surgery, but these were beyond the scope of the Original Article. Furthermore, decarbonization of operating theatres is essential because, as the current large global unmet need for surgery is addressed over the coming years, the overall volume of surgery will substantially increase.

While the literature review mainly captured interventions related to anaesthesia, the global consultation survey identified many potential interventions relating to wider processes in theatre (see the Supplementary material for the Original Article). Many of these proposed interventions are at an early stage and require robust evaluation. Governments are likely to prioritize sustainable healthcare, and high-quality, robust studies are needed to create an effective change in practice.

Education and carbon literacy are undoubtedly crucial to achieving behavioural change and implementation of environmentally sustainable interventions in healthcare. In the Original Article, healthcare professionals were invited to score the feasibility and perceived safety of shortlisted interventions, rather than their effectiveness in reducing carbon impact. Carbon modelling will be required to further prioritize the feasible interventions and this should then be validated during real-world implementation.
